# Integrating bulk and single-cell RNA data with machine learning to explore mitophagy in periodontitis

**DOI:** 10.1097/MD.0000000000044002

**Published:** 2025-08-22

**Authors:** Qisheng Hu, Yongheng Zhang, Huawei Ming, Zongyi Yuan, Fangyuan Chen, Wenjie Hao, Xiaoyao Tan, Xingan Zhang

**Affiliations:** a Department of Stomatology, North Sichuan Medical College, Nanchong, Sichuan, China; b Department of Oral and Maxillofacial Surgery, Beijing Anzhen Nanchong Hospital of Capital Medical University, Nanchong Central Hospital, The Second Clinical Medical College of North Sichuan Medical College, Nanchong, Sichuan, China.

**Keywords:** BNIP3L, CTTN, MAP1LC3B, mitophagy, periodontitis, single-cell RNA sequencing, VPS13C

## Abstract

Periodontitis (PD) is a chronic inflammatory disease in which oxidative stress plays a crucial role in its progression. Mitophagy eliminates damaged mitochondria and alleviates oxidative stress; however, its specific regulatory mechanisms in PD remain unclear. This study utilized single-cell and bulk RNA sequencing data to identify core genes and investigate their potential roles. We utilized single-cell RNA sequencing data and applied 4 algorithms – area under the curve cell level enrichment, *U*-statistics-based single-cell signature scoring, single-sample gene set scoring, and AddModuleScore – to assess mitophagy activity and identify candidate genes. Subsequently, based on bulk RNA-seq data, 5 machine learning algorithms, including Least Absolute Shrinkage and Selection Operator Regression, random forest, Boruta, gradient boosting machine, and eXtreme Gradient Boosting, were employed to further screen core genes from the candidate gene set. Finally, immune infiltration analysis, cell communication analysis, and gene interaction network construction were integrated to systematically elucidate the regulatory mechanisms of core genes in the progression of PD. Single-cell RNA sequencing combined with multiple algorithms revealed significantly elevated mitophagy activity in PD tissues, particularly in monocytes/macrophages and endothelial cells. Additionally, we identified 4 core genes: BNIP3L, VPS13C, CTTN, and MAP1LC3B. BNIP3L and CTTN were downregulated in periodontitis, correlating negatively with disease prevalence, immune infiltration, and inflammatory pathways, whereas VPS13C and MAP1LC3B were upregulated, showing positive correlations. CellChat analysis highlighted monocytes/macrophages and endothelial cells with high core gene expression as key mediators of intercellular communication. This study identified BNIP3L, VPS13C, CTTN, and MAP1LC3B as core mitophagy-related genes associated with PD, and highlighted the pivotal roles of monocytes/macrophages and endothelial cells in disease progression. These findings provide new insights into the pathogenesis of PD and offer a theoretical foundation for mitophagy-targeted diagnosis, biomarker identification, and precision therapy.

## 1. Introduction

Periodontitis (PD) is a chronic inflammatory oral disease primarily caused by bacterial infection, affecting periodontal tissues such as the gingiva, periodontal ligament, and alveolar bone.^[1]^ It is estimated that a significant proportion of adults worldwide are affected by PD to varying degrees, with most individuals presenting with periodontal symptoms after the age of 40, such as gingival bleeding, halitosis, and tooth mobility or loss.^[2]^ Recent studies have explored the role of growth factors like VEGF in enhancing bone regeneration and angiogenesis in PD treatment.^[3,4]^ Despite therapeutic advances, the effectiveness of current therapies continues to be limited by challenges, including difficulties in early diagnosis, poor patient adherence to treatment, systemic health conditions, and bacterial resistance.^[5]^ Consequently, further research is essential to provide a solid theoretical foundation for the development of new therapeutic strategies, aimed at addressing the complex clinical challenges posed by this condition.

Accumulating evidence indicates that the pathological progression of PD is closely associated with oxidative stress (OS).^[6]^ OS damages periodontal tissues by generating excessive reactive oxygen species (ROS) and activates various signaling pathways, which leads to the exacerbation of the inflammatory response.^[6]^ These inflammatory reactions further intensify OS, creating a vicious cycle that accelerates the progression of the disease.^[7]^ Besides being the primary energy suppliers, mitochondria are a major source of OS.^[8,9]^ Under excessive ROS stimulation, the mitochondrial membrane structure suffers oxidative damage, impairing the function of the respiratory chain, which leads to the loss of mitochondrial membrane potential and further exacerbates ROS generation.^[8,9]^ This affects the energy supply of mitochondria, while also intensifying intracellular OS. Mitophagy, a key cellular protective mechanism, selectively eliminates damaged and dysfunctional mitochondria through a specific autophagic process, thereby alleviating cellular damage. Emerging evidence suggests that mitophagy not only helps remove damaged mitochondria but also effectively reduces OS.^[10]^ By maintaining mitochondrial functional integrity, mitophagy is hypothesized to play a pivotal role in reducing OS and inflammatory responses in PD.^[11]^ Although direct evidence linking mitophagy to PD remains scarce, its regulatory potential offers promising avenues for research and therapeutic development.

With the continuous advancement of technology, single-cell RNA sequencing (scRNA-seq) has emerged as a pivotal tool for studying complex biological processes.^[12]^ Unlike bulk RNA sequencing, scRNA-seq captures the heterogeneity of individual cells, making it particularly valuable for studying the diverse cellular populations in inflamed periodontal tissues.^[13]^ In the field of bioinformatics, scRNA-seq offers robust data support for elucidating gene regulatory networks and intercellular communication patterns.^[14]^ In this study, we utilized scRNA-seq and bulk RNA-seq data to perform a comprehensive bioinformatics analysis. Through systematic bioinformatics analysis, we identified 4 mitophagy genes closely associated with PD and further explored the intercellular communication networks and key signaling pathways they are involved in. These findings lay the groundwork for novel therapeutic approaches targeting mitochondrial dysfunction in PD.

## 2. Material and methods

### 2.1. Data acquisition

**Figure 1.**
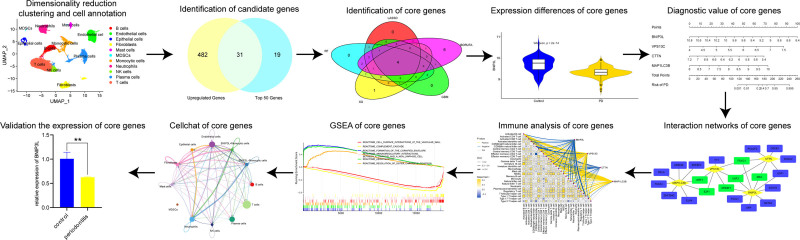
The workflow of this study.

The overall workflow of the study is illustrated in Figure 1. The scRNA-seq dataset GSE171213 and the bulk RNA-seq datasets GSE16134, GSE23586, GSE173078, and GSE223924 were retrieved from the GEO database (https://www.ncbi.nlm.nih.gov/geo). GSE171213 and GSE16134 were used as training sets, while GSE23586, GSE173078, and GSE223924 served as validation sets. The scRNA-seq dataset GSE171213 includes periodontal tissue samples from 4 healthy individuals and 5 untreated PD patients.^[15]^ The bulk RNA-seq dataset GSE16134 consisted of 310 samples, including 69 healthy individuals and 241 PD patients.^[16]^ Additionally, GSE23586 contained 3 healthy tissues and 3 PD tissues, GSE173078 comprised 12 healthy tissues and 12 PD tissues, and GSE223924 included 10 healthy tissues and 10 PD tissues (Table S1, Supplemental Digital Content, https://links.lww.com/MD/P721).^[15,17,18]^ Additionally, 71 mitophagy-related genes were obtained from the gene set enrichment analysis (GSEA) database (https://www.gsea-msigdb.org/gsea/index.jsp; Table S2, Supplemental Digital Content, https://links.lww.com/MD/P721).

### 2.2. ScRNA-seq data processing

The scRNA-seq data were processed using the Seurat package (version 4.4.0, Satija Lab at the New York Genome Center, New York),^[19]^ with each sample converted into a Seurat object. To ensure data reliability, cells with 200 to 5000 detected genes and 200 to 30,000 total RNA molecules were retained, while those with mitochondrial gene expression exceeding 30% or hemoglobin gene expression above 5% were excluded. The filtered data were normalized using the LogNormalize method with a scale factor of 10,000 to adjust for differences in sequencing depth, followed by PCA dimensionality reduction based on highly variable features,^[20]^ with the top 15 principal components selected for subsequent analyses. Batch effects across samples, defined by orig.ident, were corrected using the Harmony (version 1.2.1). The FindNeighbors function was employed to construct a *k*-nearest neighbor graph, which was further utilized by the FindClusters function to perform clustering analysis with a resolution parameter of 1.0. In the final step, cell clusters were annotated using canonical marker genes (Table S3, Supplemental Digital Content, https://links.lww.com/MD/P721), and the distribution of distinct cell subpopulations was visualized via UMAP.^[21]^

### 2.3. Assessment of mitophagy activity

In this study, 4 algorithms, namely area under the curve cell level enrichment,^[22]^ *U*-statistics-based single-cell signature scoring,^[23]^ single-sample gene set scoring,^[24]^ and AddModuleScore,^[25]^ were applied to evaluate mitophagy activity at the single-cell level based on the “GOBP_MITOPHAGY” gene set. Scoring results from all algorithms were normalized to a 0 to 1 scale, then aggregated to generate a composite score termed SumScore. Spearman correlation analysis was performed to assess the relationship between the expression levels of each gene and the SumScore. Furthermore, cells were divided into high-activity and low-activity groups based on the median SumScore. Differential gene expression analysis was performed between these 2 groups using the FindMarkers function, with the criteria set as log_2_ FC > 0 and *P*_adj_ < .05. The 513 significantly upregulated genes were intersected with the top 50 genes from the correlation analysis, resulting in 31 candidate genes. Through enrichment analysis, comprising Kyoto Encyclopedia of Genes and Genomes (KEGG)^[26]^ and Gene Ontology (GO) analysis,^[27]^ the biological significance of the candidate genes was investigated.

### 2.4. Determining core genes with machine learning algorithms

Based on the GSE16134 dataset, we employed 5 machine learning algorithms, including Least Absolute Shrinkage and Selection Operator (LASSO), random forest (RF), Boruta, gradient boosting machine (GBM), and eXtreme Gradient Boosting (XGBoost), to prioritize candidate genes and ultimately determining the core genes. LASSO was applied to select genes with nonzero coefficients by incorporating regularization and optimizing the model through 10-fold cross-validation.^[28]^ RF ranked the importance of genes based on their contributions to classification accuracy.^[29]^ Boruta identified candidate genes by contrasting random shadow variables with actual variables, employing iterative optimization to refine the selection process.^[30]^ GBM assessed the relative influence of each gene to identify significant contributors,^[31]^ while XGBoost evaluated feature importance based on gain values and implemented early stopping to mitigate overfitting.^[32]^ Finally, the core genes were determined by taking the intersection of the results from the 5 algorithms.

### 2.5. Expression differences and diagnostic value of core genes

Based on the GSE16134 dataset, expression differences of core genes between PD and healthy tissues were analyzed. Subsequently, the diagnostic performance of the core genes was evaluated by constructing receiver operating characteristic curves, with the area under the curve (AUC) serving as a quantitative metric of diagnostic accuracy.^[33]^ Furthermore, a Nomogram incorporating multiple core genes was developed to predict the risk of PD by quantifying gene expression levels. The predictive accuracy and clinical utility of the Nomogram were rigorously validated through calibration plot analysis and decision curve analysis (DCA), underscoring its potential clinical relevance.^[34]^

### 2.6. Construction of interaction networks of core genes

To systematically analyze the regulatory mechanisms of core genes, the NetworkAnalyst platform (https://www.networkanalyst.ca/) was utilized to construct interaction networks.^[35]^ A transcription factor (TF)-gene regulatory network was established based on the JASPAR database to predict upstream TFs that potentially regulating core gene expression. The gene-miRNA regulatory network was constructed using the miRTarBase v9.0 database to identify miRNA interactions associated with core genes. Furthermore, the STRING database (confidence score ≥ 700) was employed to construct a protein–protein interaction (PPI) network, providing insights into protein-level interactions and potential functional modules of the core genes.

### 2.7. Immune infiltration and immune pathway analysis

To explore the differences in immune infiltration between PD and normal tissues and investigate the immune associations of core genes, a multilevel analysis was conducted based on the GSE16134 dataset and predefined immune marker gene sets (Table S4, Supplemental Digital Content, https://links.lww.com/MD/P721). The single-sample gene set enrichment analysis (ssGSEA) algorithm was employed to determine immune cell infiltration levels in PD and normal tissues. The correlation between the expression levels of core genes and immune cell infiltration was evaluated to determine potential associations.^[36]^ Furthermore, ssGSEA was applied to compute enrichment scores for a custom immune-related gene set (Table S5, Supplemental Digital Content, https://links.lww.com/MD/P721), and the correlations between core genes and critical immune pathways were analyzed to elucidate potential immune regulatory mechanisms.

### 2.8. GSEA enrichment analysis

To further investigate the potential biological functions of the core genes and their associated signaling pathways, GSEA was conducted leveraging the GSE16134 dataset and the c2.cp.reactome.v7.0 collection from MSigDB (Table S6, Supplemental Digital Content, https://links.lww.com/MD/P721).^[37]^

### 2.9. Cell communication analysis

To explore the potential regulatory roles of core genes in PD associated cells and their mechanisms of intercellular communication, cell communication analysis was conducted using the CellChat package (version 1.6.1, University of California, Irvine) based on PD samples from the GSE171213 dataset.^[14]^ The expression differences of each core gene across specific cell types were analyzed, and the cell type with the highest expression was selected for further study. This cell type was divided into high-expression and low-expression groups according to the expression levels of the core gene. Communication networks between the high- and low-expression groups and their interactions with other cell types were inferred using the Secreted Signaling database in CellChat. Additionally, ligand–receptor interactions were predicted by integrating the PPI network to evaluate the strength of interactions. Finally, KEGG enrichment analysis was performed on differentially expressed genes identified in the high-expression group.

### 2.10. Cell culture and periodontitis model establishment

All reagents, drugs, cell lines, and their respective commercial suppliers are comprehensively listed in Table S17, Supplemental Digital Content, https://links.lww.com/MD/P721. Human gingival fibroblasts, generously gifted by North Sichuan Medical College, were utilized in this study for cell culture and experimental analysis. Primary culture cells were ethically sourced under Nanchong Central Hospital Approval No. 025 (2025). The cells were cultured in Dulbecco’s Modified Eagle Medium supplemented with 10% fetal bovine serum and 1% penicillin-streptomycin under standard conditions of 37°C and 5% CO₂ in a humidified atmosphere. To establish an in vitro PD model, cells were treated with lipopolysaccharide (LPS) at a concentration of 1 µg/mL for 6 hours. Samples were collected after treatment for subsequent analyses.

### 2.11. Real time quantitative PCR (RT-qPCR)

Total RNA was extracted from cell samples using the TRIzol reagent (No. 9109, TaKaRa, Japan), and reverse transcription was performed using the PrimeScript RT Kit (No. RR036A, TaKaRa, Japan). The resulting cDNA was quantified using UltraSYBR Mixture (CW0957H, CWBIO, China). GAPDH served as the internal control, and relative expression levels were calculated using the 2^−ΔΔCt^ method. All experimental procedures were conducted in strict accordance with the manufacturer’s protocols, with each sample analyzed in triplicate to ensure accuracy and reliability. The sequences of the primers used in this study are as follows: BNIP3L forward 5′-ATGTCGTCCCACCTAGTCGAG-3′ and reverse 5′-TGAGGATGGTACGTGTTCCAG-3′, VPS13C forward 5′-TGTGGAAAAATTGGCAACTCAAG-3′ and reverse 5′-CCCAGTGTGACACCAAATGAA-3′, CTTN forward 5′-GCTTTGAGTATCAAGGCAAAACG-3′ and reverse 5′-CCAAGGGCACATTTGTCTTGT-3′, MAP1LC3B forward 5′-AAGGCGCTTACAGCTCAATG-3′ and reverse 5′-CTGGGAGGCATAGACCATGT-3′, and GAPDH forward 5′-GGAGTCCACTGGCGTCTTCA-3′ and reverse 5′-GTCATGAGTCCTTCCACGATACC-3′.

### 2.12. Statistical analysis

Data processing and analysis were performed using R software (version 4.2.2). The Venn diagram for the selection of candidate genes was generated using the bioinformatics website (http://bioinformatics.com.cn/). Group differences in quantitative PCR (qPCR) data were evaluated using *t*-tests, and the results were reported as mean ± standard error of the mean. Graphical representations of qPCR data were generated using GraphPad Prism 8.0. Interaction network visualization was conducted using Cytoscape_v3.10.3. A *P*-value of less than .05 was considered statistically significant in all analyses.

## 3. Results

### 3.1. Dimensionality reduction clustering and cell annotation

**Figure 2.**
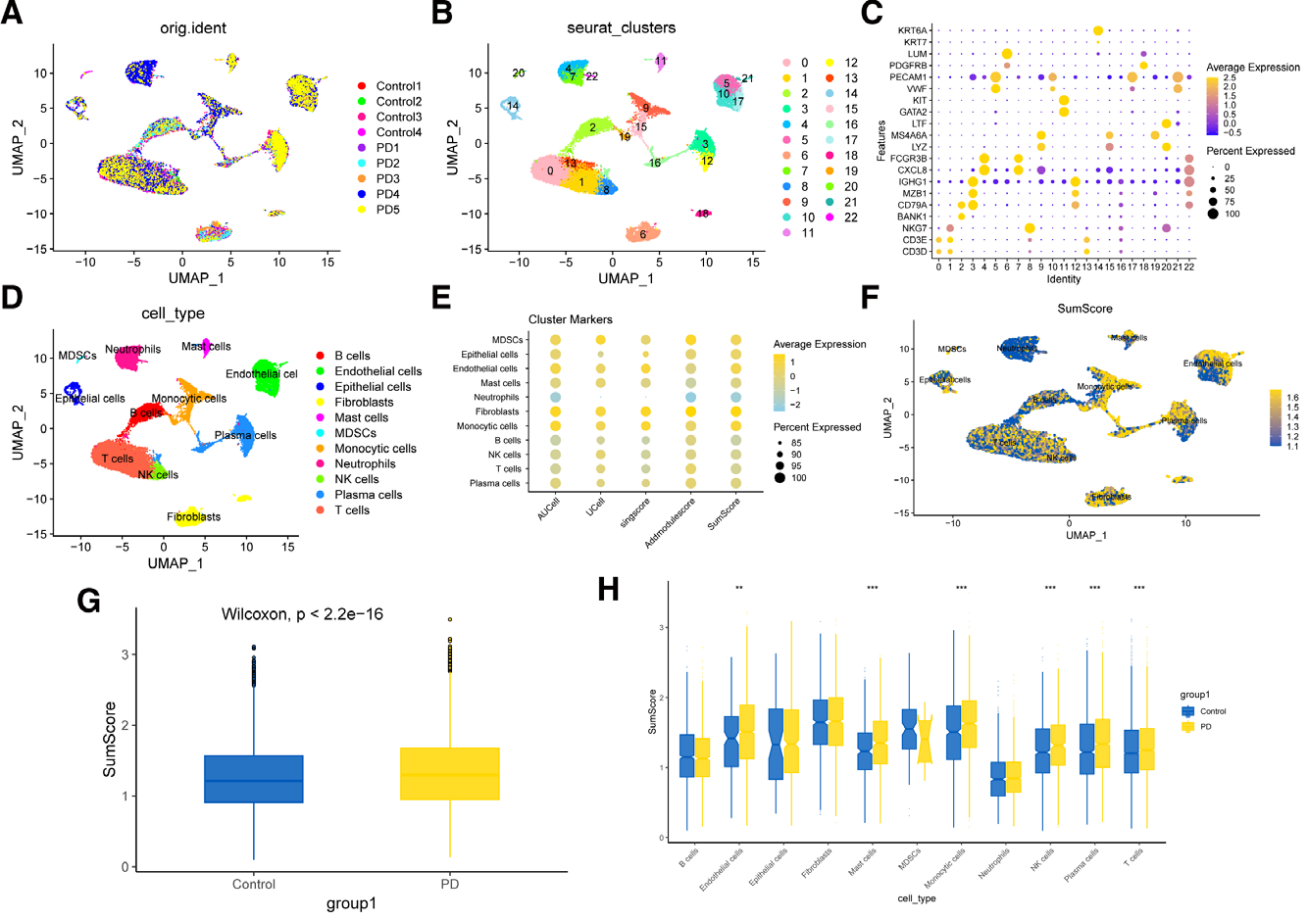
Dimensionality reduction clustering and cell annotation. (A) PCA analysis shows a high consistency in the distribution of all cells, with no significant batch effects observed. (B) Based on the heterogeneity of cell types, all cells were successfully classified into 22 clusters. (C, D) Using canonical cell marker genes, all cells were classified and manually annotated. (E) Expression of mitophagy-related genes in different cell clusters evaluated by various algorithms. (F) Spatial distribution of SumScore values across different cell types. (G, H) SumScore in the periodontitis group is significantly higher than in the control group, particularly in mast cells, monocytic cells, plasma cells, NK cells, and T cells. PCA = principal component analysis.

The PCA analysis results demonstrated a high degree of consistency in the cellular distribution across both PD and normal tissue samples, with no significant technical biases detected following batch effect correction (Fig. 2A). UMAP analysis further revealed that all cells were successfully clustered into 22 distinct groups, with the distribution of these clusters closely reflecting the cellular heterogeneity (Fig. 2B). By classifying the cell clusters based on the expression of canonical marker genes, we manually annotated the cell types, accurately identifying a variety of cell populations, including T cells, NK cells, B cells, plasma cells, neutrophils, monocytic cells, MDSCs, mast cells, endothelial cells, fibroblasts, and epithelial cells, ensuring accurate classification of each population (Fig. 2C, D). To investigate the activity of mitophagy in PD, we employed algorithms such as area under the curve cell level enrichment, *U*-statistics-based single-cell signature scoring, single-sample gene set scoring, AddModuleScore to assess the expression of mitophagy-related genes in each cell. The results revealed that gene expression displayed heterogeneity across different cell clusters, while the scoring results from various algorithms demonstrated a high degree of consistency (Fig. 2E). Through UMAP visualization, we illustrated the distribution of SumScore values across distinct cell types, revealing significant aggregation in monocytic cells, endothelial cells, plasma cells and fibroblasts (Fig. 2F). Further boxplot analysis revealed that the SumScore in the PD group was significantly higher than in the control group (*P* < .001, Wilcoxon test), with notably higher in endothelial cells, mast cells, monocytic cells, plasma cells, NK cells, and T cells (Fig. 2G, H).

### 3.2. Identification of candidate genes

**Figure 3.**
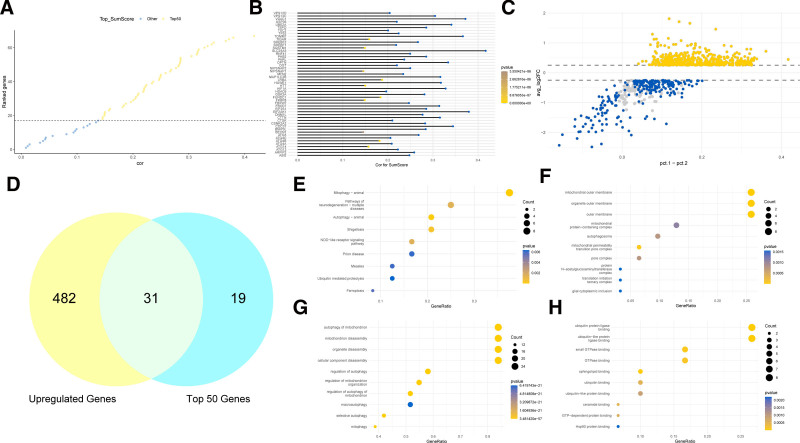
Identification of candidate genes. (A, B) Top 50 genes most strongly correlated with SumScore based on Spearman correlation analysis. (C) Differential expression analysis results between the SumScore Up and SumScore Down groups. (D) Intersection of significantly upregulated genes from differential analysis and the top 50 genes from correlation analysis, resulting in 31 candidate genes. (E) KEGG enrichment analysis of the candidate genes. (F) GO cellular component enrichment analysis of the candidate genes. (G) GO biological process enrichment analysis of the candidate genes. (H) GO molecular function enrichment analysis of the candidate genes. GO = Gene Ontology, KEGG = Kyoto Encyclopedia of Genes and Genomes.

Based on the Spearman correlation analysis between SumScore and gene expression, we identified the top 50 genes most strongly correlated with SumScore (Fig. 3A, B). Subsequently, using the median SumScore, we divided the single-cell data into SumScore Up and SumScore Down groups and conducted differential expression analysis between them (Fig. 3C). From the analysis, we identified 513 significantly upregulated genes, which were intersected with the top 50 genes from the Spearman correlation analysis, yielding 31 candidate genes (Fig. 3D, Table S7, Supplemental Digital Content, https://links.lww.com/MD/P721). These candidate genes were then subjected to KEGG and GO enrichment analyses. The results showed that: in the KEGG pathway analysis, the candidate genes were significantly enriched in mitophagy – animal and pathways of neurodegeneration – multiple diseases; in the GO molecular function analysis, they were significantly enriched in ubiquitin protein ligase binding and ubiquitin-like protein ligase binding; in the GO cellular component analysis, they were significantly enriched in mitochondrial outer membrane, organelle outer membrane, and outer membrane; and in the GO biological process analysis, they were significantly enriched in autophagy of mitochondrion and mitochondrion disassembly (Fig. 3E–H).

### 3.3. Core gene selection via multiple machine learning algorithms

**Figure 4.**
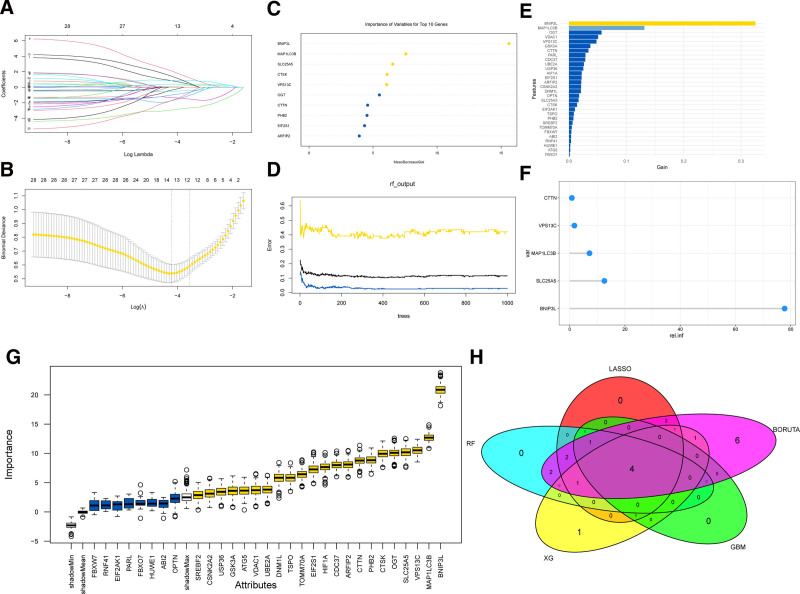
Core gene selection via multiple algorithmic approaches. (A) Plot of the LASSO coefficient path. As lambda increases, the coefficients of less important features shrink to 0. (B) Plot of cross-validation error in LASSO regression, showing the relationship between log(λ) and binomial deviance. (C) Top 10 genes identified by the random forest (RF) algorithm that significantly contribute to distinguishing sample categories. (D) Error rate plot for the random forest (RF) model, showing how the error rate changes with the number of trees. (E) Feature importance plot for XGBoost, showing the genes ranked by their importance scores (gain). (F) Top 5 genes identified by the GBM model based on their relative importance. (G) Feature importance plot for the Boruta algorithm, showing the identification of 22 important genes after ranking. (H) Venn diagram showing the intersection of core genes identified by LASSO, RF, XGBoost, GBM, and Boruta algorithms. GBM = gradient boosting machine, LASSO = Least Absolute Shrinkage and Selection Operator, RF = random forest, XGBoost = eXtreme Gradient Boosting.

We employed the GSE16134 dataset in combination with 5 algorithms to identify core genes. Through training and cross-validation of the LASSO regression model, we identified 13 genes significantly associated with the target variable, which played a pivotal role in the binary classification model (Fig. 4A, B, Table S8, Supplemental Digital Content, https://links.lww.com/MD/P721). By applying the RF algorithm for training and variable importance analysis, we identified 10 genes that made significant contributions to distinguishing sample categories (Fig. 4C, D, Table S9, Supplemental Digital Content, https://links.lww.com/MD/P721). Using XGBoost, we ranked the genes based on their importance scores and selected the top 10 for subsequent analysis (Fig. 4E, Table S10, Supplemental Digital Content, https://links.lww.com/MD/P721). Through the GBM algorithm, we identified 5 genes with the highest contribution to the binary classification task (Fig. 4F, Table S11, Supplemental Digital Content, https://links.lww.com/MD/P721). Feature selection using the Boruta algorithm identified 22 genes closely associated with the target variable (Fig. 4G, Table S12, Supplemental Digital Content, https://links.lww.com/MD/P721). To validate the stability and consistency of these genes, we performed an intersection analysis of the results from the LASSO, RF, XGBoost, Boruta, and GBM algorithms, ultimately identifying 4 core genes: BNIP3L, VPS13C, CTTN, and MAP1LC3B (Fig. 4H).

### 3.4. Expression differences and diagnostic value of core genes

**Figure 5.**
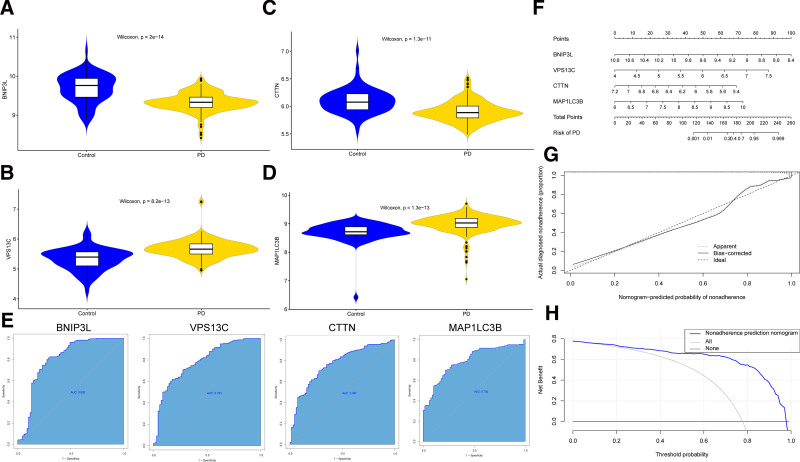
Expression differences and diagnostic value of core genes. (A–D) Violin plots showing the expression of core genes in healthy and periodontitis tissues. (E) ROC curves depicting the diagnostic performance of core genes in distinguishing healthy and periodontitis tissues. (F) Nomogram for predicting the risk of periodontitis based on the expression of core genes. (G) Calibration plot for the Nomogram model to evaluate the predictive accuracy. (H) Decision curve analysis (DCA) for the Nomogram model to evaluate its net benefit at different threshold probabilities. DCA = decision curve analysis, ROC = receiver operating characteristic.

To investigate the expression differences of the core genes BNIP3L, CTTN, VPS13C, and MAP1LC3B between healthy and PD tissues, we analyzed the GSE16134 dataset and generated violin plots for these genes (Fig. 5A–D). The results indicated that, in comparison to healthy tissues, the expression of BNIP3L and CTTN was significantly lower in PD tissues, while the expression of VPS13C and MAP1LC3B was notably higher (Wilcoxon test, *P* < .05). To assess the diagnostic ability of these core genes, we constructed receiver operating characteristic curves and calculated AUC (Fig. 5E). The results demonstrated that all core genes exhibited strong discriminatory power between healthy tissues and PD, with AUC values >0.7, indicating their excellent diagnostic performance. To further evaluate the potential of these core genes in predicting PD risk, we constructed a Nomogram model to estimate risk based on gene expression (Fig. 5F). The results showed that, as the expression levels of BNIP3L and CTTN increased, the risk of PD decreased, whereas higher expression levels of VPS13C and MAP1LC3B were associated with an increased risk of PD. To evaluate the predictive accuracy of the Nomogram model, we used a calibration plot to compare predicted risk probabilities with actual outcomes (Fig. 5G). The bias-corrected model closely aligned with the ideal line, indicating good predictive performance. Additionally, we employed DCA to assess the net benefit of the model at different thresholds (Fig. 5H). The net benefit of the Nomogram model was significantly higher than that of the “All” and “None” strategies in DCA, suggesting that the model has high clinical utility in decision-making.

### 3.5. Interaction networks of core genes

**Figure 6.**
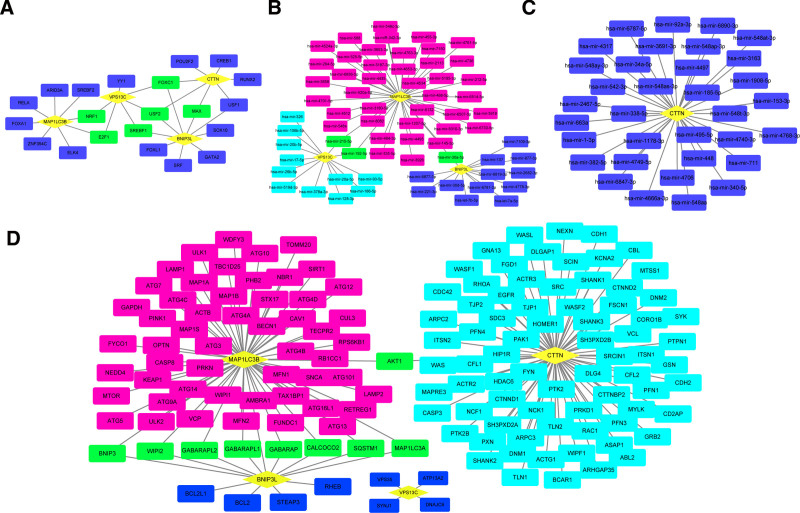
Interaction networks of core genes. (A) Transcription factor-gene (TF-gene) regulatory network of core genes. (B, C) Gene-miRNA regulatory network of core genes. (D) Protein–protein interaction (PPI) network of core genes. TF = transcription factor.

To further explore the multilevel regulatory mechanisms of core genes, we utilized the NetworkAnalyst platform and constructed TF-gene regulatory networks based on the JASPAR database (Fig. 6A). The results demonstrated that MAP1LC3B and VPS13C were co-regulated by the TFs NRF1 and E2F1. VPS13C and BNIP3L shared the TF SREBF1, while BNIP3L and CTTN shared the TF MAX. The TFs USF2 and FOXC1 were found to jointly regulate the 3 core genes BNIP3L, CTTN, and VPS13C. Subsequently, gene-miRNA regulatory networks were constructed using the miRTarBase v9.0 database (Fig. 6B, C). The analysis revealed that VPS13C and MAP1LC3B might be co-regulated by miRNAs hsa-mir-215-5p and hsa-mir-192-5p, while BNIP3L and MAP1LC3B might be co-regulated by hsa-mir-30a-5p. In addition, we utilized the STRING database to construct PPI networks for the core genes (Fig. 6D). The analysis revealed that BNIP3L and MAP1LC3B interacted with multiple key proteins, including BNIP3, WIPI2, GABARAPL2, GABARAPL1, GABARAP, CALCOCO2, SQSTM1, and MAP1LC3A, while MAP1LC3B and CTTN were found to interact with the protein AKT1. These findings indicate that core genes are closely interconnected and likely participate in the regulation of related signaling pathways across multiple regulatory levels.

### 3.6. Immune infiltration and immune pathway analysis

**Figure 7.**
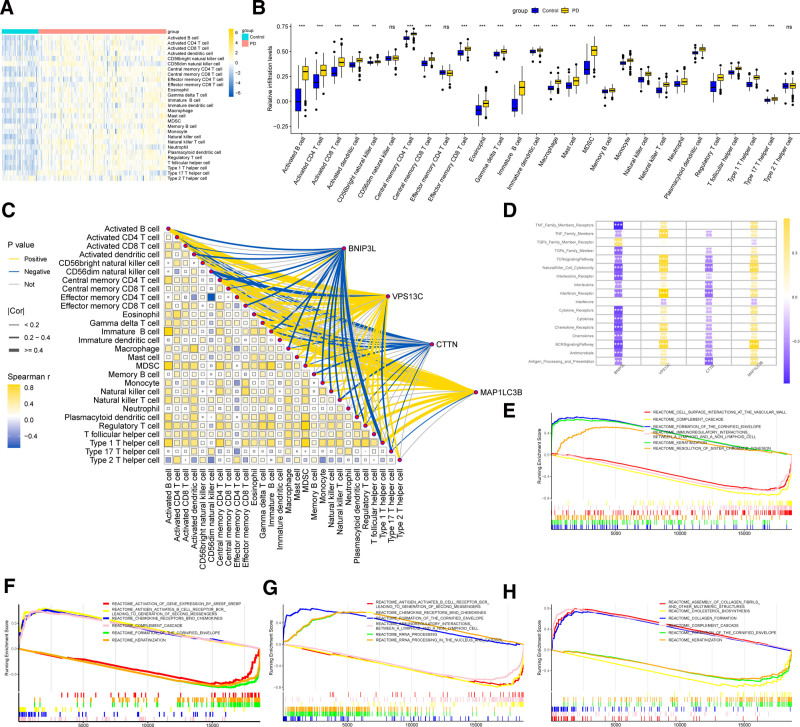
Immune Infiltration and GSEA of core genes. (A, B) The relative infiltration levels of different immune cells in periodontitis and healthy tissues. (C) Spearman correlation between core genes and various immune cell infiltration levels, as well as the correlations among different immune cell types. (D) Spearman correlation between core genes and key immune pathways. (E) GSEA results for BNIP3L. (F) GSEA results for VPS13C. (G) GSEA results for CTTN. (H) GSEA results for MAP1LC3B. GSEA = gene set enrichment analysis.

Using the ssGSEA method and predefined immune marker gene sets, we analyzed the relative infiltration levels of various immune cell types in PD and healthy tissues. The results demonstrated that, with the exception of CD56dim natural killer cells, effector memory CD4 T cells, and type 2 T helper cells, most immune cells exhibited significantly higher infiltration levels in PD tissues compared to healthy (Fig. 7A, B). This indicates that immune-related activity is significantly higher in PD tissues, potentially influenced by the inflammatory microenvironment. Furthermore, we calculated the Spearman correlations between core genes and immune cell infiltration levels, as well as the interrelationships among various immune cell types in PD tissues (Fig. 7C). The analysis revealed that VPS13C and MAP1LC3B showed significant correlations with the majority of immune cells. Additionally, activated B cells, myeloid-derived suppressor cells, regulatory T cells, central memory CD4 T cells, activated dendritic cells, and type 1 T helper cells displayed strong correlations with the infiltration levels of various immune cell types. Using a custom-defined immune pathway gene set in ssGSEA, we calculated the Spearman correlations between core genes and critical immune pathways (Fig. 7D). The results showed that VPS13C and MAP1LC3B exhibited significant positive correlations with the majority of immune pathways, whereas BNIP3L and CTTN were negatively correlated. These findings suggest that VPS13C and MAP1LC3B may enhance immune activity, contributing to the chronic inflammation and immune dysregulation observed in PD.

### 3.7. The results of GSEA for core genes

Based on the Reactome gene set from MSigDB, GSEA was conducted to evaluate the distribution of core genes across Reactome pathways. The results revealed that BNIP3L exhibited significant enrichment in the formation of the cornified envelope, keratinization, and resolution of sister chromatid cohesion pathways, while showing the lowest enrichment in cell surface interactions at the vascular wall, immunoregulatory interactions between a lymphoid and a nonlymphoid cell, and complement cascade pathways (Fig. 7E, Table S13, Supplemental Digital Content, https://links.lww.com/MD/P721). VPS13C demonstrated significant enrichment in complement cascade, chemokine receptors bind chemokines, and antigen activates B cell receptor BCR leading to generation of second messengers pathways, but showed minimal enrichment in formation of the cornified envelope, keratinization, and activation of gene expression by SREBF (SREBP) pathways (Fig. 7F, Table S14, Supplemental Digital Content, https://links.lww.com/MD/P721). CTTN was significantly enriched in formation of the cornified envelope, rRNA processing, and rRNA processing in the nucleus and cytosol pathways, with the lowest enrichment observed in chemokine receptors bind chemokines, immunoregulatory interactions between a lymphoid and a nonlymphoid cell, and antigen activates B cell receptor BCR leading to generation of second messengers pathways (Fig. 7G, Table S15, Supplemental Digital Content, https://links.lww.com/MD/P721). Additionally, MAP1LC3B showed strong enrichment in assembly of collagen fibrils and other multimeric structures, complement cascade, and collagen formation pathways, while exhibiting the lowest enrichment in formation of the cornified envelope, keratinization, and cholesterol biosynthesis pathways (Fig. 7H, Table S16, Supplemental Digital Content, https://links.lww.com/MD/P721). These analyses underscore the diverse roles of these core genes in various biological processes and highlight their differential involvement across Reactome pathways.

### 3.8. CellChat of core genes in periodontitis

**Figure 8.**
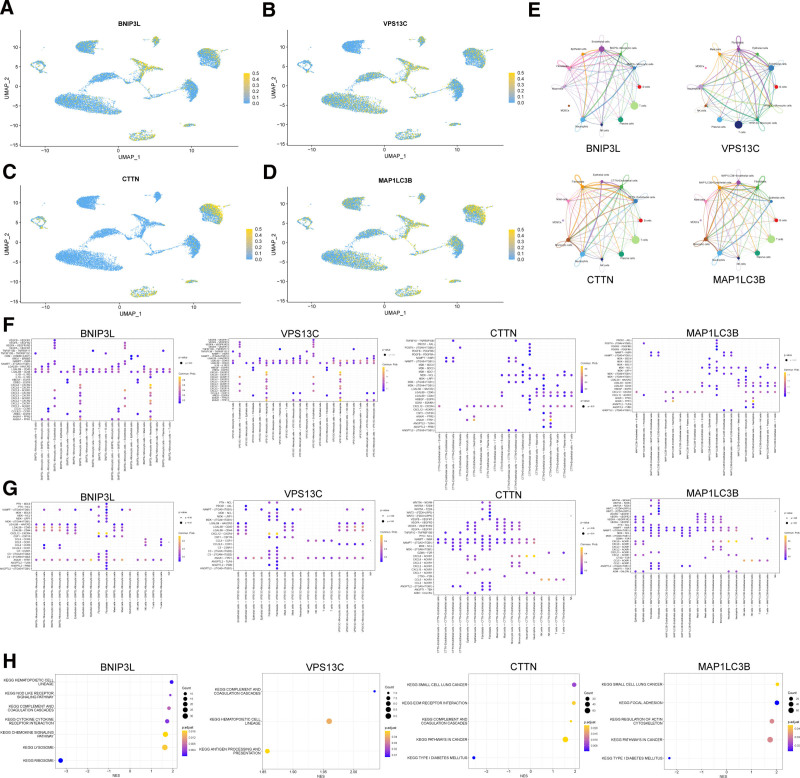
CellChat of core genes in periodontitis. (A–D) Expression profiles of core genes in different cell types within periodontal tissue. (E) Count of communication interactions between the core gene and other cell types after categorizing them into “core gene + cell type” and “core gene − cell type.” (F, G) Signal pathways and the probability of cell–cell communication after core gene grouping. (H) GSEA results of differentially expressed genes after core gene grouping. GSEA = gene set enrichment analysis.

Based on preprocessed scRNA-seq data, we analyzed the expression profiles of core genes across different cell types in PD tissues. The results revealed that BNIP3L and VPS13C were predominantly expressed in monocytic cells, whereas CTTN and MAP1LC3B were highly expressed in endothelial cells (Fig. 8A–D). The cell type with the highest expression of each core gene was identified, and cells were classified as “core gene + cell type” or “core gene − cell type” based on the presence or absence of gene expression. Cell–cell communication analysis was performed on these 2 groups, and circle plots illustrated their communication counts with other cell types (Fig. 8E).

The bubble plot was further used to visualize the signaling pathways within the cell–cell communication network, with communication probability represented visually (Fig. 8F, G). The results indicated that when BNIP3L + and BNIP3L − Monocytic cells acted as signaling sources, BNIP3L − cells exhibited higher communication probability exclusively in the VEGFB − VEGFR1 pathway with endothelial cells. In contrast, BNIP3L + Monocytic cells showed higher communication probabilities across various target cell types, including the VEGFA − VEGFR2, VEGFA − VEGFR1R2, VEGFA − VEGFR1, NAMPT − (ITGA5 + ITGB1), NAMPT − INSR, CXCL8 − ACKR1, CXCL3 − ACKR1, and CXCL2 − ACKR1 pathways with endothelial cells; the OSM − (OSMR + IL6ST) pathways in epithelial cells and fibroblasts; the IL1A − IL1R2, CXCL8 − CXCR2, CXCL8 − CXCR1, CXCL3 − CXCR2, CXCL3 − CXCR1, CXCL2 − CXCR1, CXCL2 − CXCR2, and ANXA1 − FPR1 pathways with neutrophils. When targeted by signals, BNIP3L + Monocytic cells also displayed higher communication probabilities, specifically in the NAMPT − (ITGA5 + ITGB1), ANXA1 − FPR1, CCL4 − CCR5, and CCL3L3 − CCR1 pathways from themselves. Additionally, the NAMPT − (ITGA5 + ITGB1) and ANXA1 − FPR1 pathways from epithelial cells, endothelial cells, and mast cells; the PTN − SDC4, NAMPT − (ITGA5 + ITGB1), MDK − SDC4, MDK − LRP1, MDK − (ITGA4 + ITGB1), C3 − C3AR1, ANXA1 − FPR1, ANGPTL2 − TLR4, ANGPTL2 − PIRB, and ANGPTL2 − (ITGA5 + ITGB1) pathways from fibroblasts; the NAMPT − (ITGA5 + ITGB1) pathway from neutrophils; and the CCL5 − CCR5, CCL5 − CCR1, CCL4 − CCR5, and ANXA1 − FPR1 pathways from NK and T cells.

When VPS13C + Monocytic cells and VPS13C − Monocytic cells acted as signaling sources, VPS13C + Monocytic cells exhibited higher communication probabilities, including LGALS9 − CD45 and LGALS9 − CD44 pathways in B cells, mast cells, neutrophils, NK cells, plasma cells, T cells, themselves; the VEGFB − VEGFR1, VEGFA − VEGFR2, VEGFA − VEGFR1R2, and VEGFA − VEGFR1 pathways in endothelial cells. When they were targeted by signals, VPS13C + Monocytic cells also exhibited higher communication probabilities. These included the NAMPT − (ITGA5 + ITGB1) pathway from epithelial cells, mast cells, endothelial cells, fibroblastic cells, neutrophils, and themselves; and the CCL5 − CCR1 pathway from NK cells and T cells.

When CTTN + Endothelial cells and CTTN − Endothelial cells acted as signaling sources, CTTN + Endothelial cells exhibited higher communication probabilities. These included the MDK − NCL, MDK − (ITGA6 + ITGB1), and ANGPTL2 − (ITGA5 + ITGB1) pathways in themselves; the MDK − NCL, LGALS9 − CD45, and LGALS9 − CD44 pathways in B cells, mast cells, NK cells, T cells, and plasma cells; the MDK − SDC4, MDK − SDC1, MDK − NCL, MDK − (ITGA6 + ITGB1), and LGALS9 − CD44 pathways in epithelial cells; the PROS1 − AXL, PDGFB − PDGFRB, PDGFB − PDGFRA, MDK − SDC2, MDK − NCL, MDK − LRP1, LGALS9 − CD44, HBEGF − EGFR, and EDN1 − EDNRA pathways in fibroblasts; the MDK − NCL, MDK − LRP1, MDK − (ITGA4 + ITGB1), LGALS9 − HAVCR2, LGALS9 − CD45, LGALS9 − CD44, CSF3 − CSF3R, and ANGPTL2 − PIRB pathways in monocytic cells; and the LGALS9 − CD45, LGALS9 − CD44, CSF3 − CSF3R, ANGPTL2 − TLR4, and ANGPTL2 − PIRB pathways in neutrophils. When they acted as signaling targets, CTTN + Endothelial cells also exhibited higher communication probabilities. These included the TNFSF10 − TNFRSF10B pathway from epithelial cells, mast cells, and themselves; the GZMA − F2R pathway from NK cells and T cells; and the WNT5A − MCAM, WNT5A − FZD6, WNT5A − FZD4, WNT2 − (FZD6 + LRP5), and WNT2 − (FZD4 + LRP5) pathways from fibroblasts.

When MAP1LC3B + Endothelial cells and MAP1LC3B − Endothelial cells acted as signaling sources, MAP1LC3B + Endothelial cells also exhibited higher communication probabilities, which were nearly identical to those observed with the CTTN gene. When they acted as signaling targets, MAP1LC3B + Endothelial cells were also largely consistent with CTTN, except for no significant differences observed in epithelial cells and higher communication probabilities in the CTSG − F2R pathway from mast cells.

Finally, we employed GSEA analysis based on the KEGG pathway database to investigate the differentially expressed genes between the “core gene + cell type” and “core gene − cell type” groups (Fig. 8H).

### 3.9. Validation of differential expression of core genes

**Figure 9.**
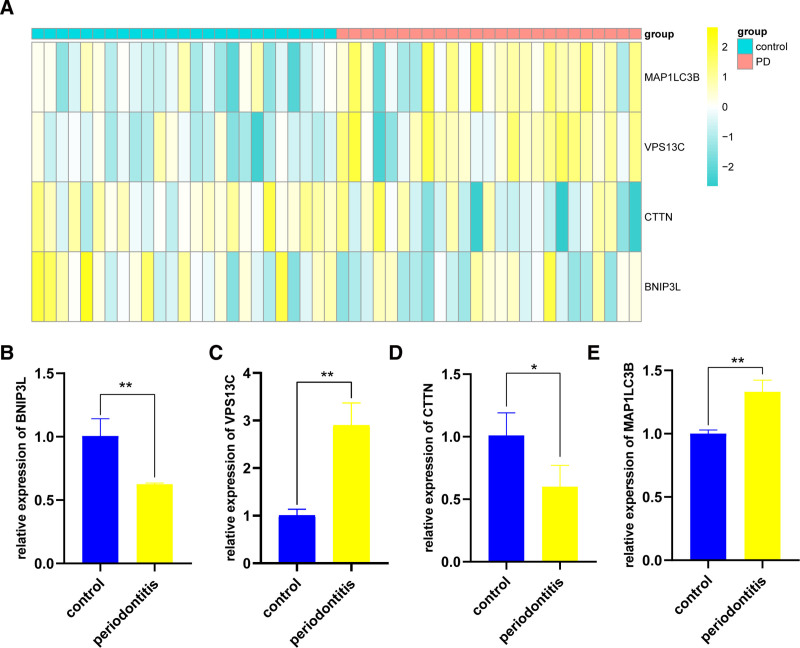
Validation of differential expression of core genes. (A) Expression of core genes in the validation set (GSE173078, GSE223924, and GSE23586). (B–E) qPCR results of core genes in periodontal fibroblasts and their LPS-induced inflammatory cell model. qPCR = quantitative PCR.

We integrated data from multiple datasets (GSE173078, GSE223924, and GSE23586) and applied batch effect correction to the expression matrices. A heatmap was employed to visualize the expression patterns of core genes across distinct groups (Fig. 9A). The results demonstrated that the expression levels of BNIP3L and CTTN were significantly higher in healthy tissues compared to PD tissues, while the expression levels of VPS13C and MAP1LC3B were markedly elevated in PD tissues relative to healthy tissues. To further validate the differential expression patterns of these core genes, we utilized human gingival fibroblasts and an LPS-induced inflammatory cell model, followed by qPCR to assess the expression levels of these genes (Fig. 9B–E). The results showed that BNIP3L and CTTN were expressed at higher levels in normal fibroblasts, while VPS13C and MAP1LC3B were significantly upregulated in the LPS-induced inflammation. The findings from the validation set and qPCR were highly consistent with the differential expression patterns observed in the training set, further validating the reliability of the results.

## 4. Discussion

PD is a chronic inflammatory disease caused by bacterial infection, primarily affecting the periodontal tissues, with symptoms worsening progressively with age.^[1]^ Despite ongoing advancements in current therapeutic approaches, their efficacy remains limited, necessitating the development of novel treatment strategies. Mitophagy, the process by which cells remove damaged mitochondria, plays a crucial role in alleviating oxidative damage and maintaining cellular homeostasis.^[38]^ Mitochondrial dysfunction is closely linked to the pathogenesis of PD,^[39]^ particularly as mitophagy may have a significant role in reducing OS and inflammatory responses. Recent studies by Jang et al have shown that the loss of PINK1 leads to impaired mitophagy, which subsequently exacerbates bone loss and the excessive differentiation of osteoclasts induced by PD.^[11]^

In this study, we observed that mitophagy activity was highest in monocytic/macrophage cells and endothelial cells in periodontal tissues, with significantly higher activity in PD tissues compared to healthy tissues. Numerous studies have confirmed the close association between monocytic/macrophage and endothelial cells with mitophagy. Patoli et al demonstrated that inhibition of mitophagy under LPS/IFN-γ stimulation promotes classical macrophage activation and increases mitochondrial ROS production.^[40]^ But there are no studies have yet explored the relationship between periodontal endothelial cells and mitophagy. Using multiple algorithms, we ultimately identified 4 core genes (BNIP3L, VPS13C, CTTN, and MAP1LC3B) that are closely associated with mitophagy in PD. We found that these core genes were similarly most actively expressed in monocytic/macrophage cells and endothelial cells. Further cell–cell communication analysis revealed that monocytic/macrophage and endothelial cells expressing the core genes play crucial roles in signaling interactions with other cells.

BNIP3L (BCL-2 Interacting Protein 3 Like), also known as NIX, is a member of the BCL-2 protein family and plays a critical role in regulating mitophagy and autophagy.^[41]^ Through its BH3-only domain, BNIP3L interacts with BCL-2 and BCL-XL, inhibiting their antiapoptotic effects and thereby promoting autophagy.^[42]^ Emerging evidence suggests that BNIP3L is significantly involved in the pathogenesis of PD, particularly in the regulation of mitophagy and apoptosis. A study by Song et al demonstrated that hyperglycemia exacerbates cellular senescence and inflammation in periodontal tissues by increasing ROS and inhibiting BNIP3L-mediated mitophagy.^[43]^ CTTN (Cortactin) is an actin-binding protein that plays a pivotal role in cytoskeletal remodeling and is involved in various cellular processes, including cell migration, invasion, endocytosis, macropinocytosis, and signal transduction.^[44]^ Bandela et al demonstrated that CTTN may mitigate OS damage by inhibiting mitochondrial ROS production, and it may also regulate mitochondria-mediated apoptotic pathways by modulating mitochondrial membrane permeability.^[45]^ However, research on its involvement in mitophagy in PD remains scarce. Our study revealed that, in periodontal tissues, BNIP3L and CTTN expression is downregulated due to the influence of the inflammatory microenvironment. This suppression may lead to impaired mitophagy, resulting in the accumulation of dysfunctional mitochondria and OS, thereby exacerbating inflammation and tissue destruction. Immunoinfiltration analysis further demonstrated a negative correlation between BNIP3L and CTTN expression and immune cell infiltration, reinforcing the association between their downregulation and immune hyperactivation in PD. Furthermore, the Nomogram analysis indicated that increased expression of BNIP3L and CTTN may contribute to reduce disease susceptibility. This study highlights the protective roles of BNIP3L and CTTN in PD, suggesting their potential as therapeutic targets for the disease and offering novel intervention strategies for modulating mitochondrial stability and inflammatory responses.

VPS13C (Vacuolar Protein Sorting 13 Homolog C) is a member of the VPS13 family, primarily involved in mitochondrial homeostasis and vesicular transport, and is closely associated with neurodegenerative and metabolic disorders.^[46]^ Mutations in VPS13C have been implicated in Parkinson’s disease and other metabolic diseases. Jia et al reported that VPS13C is downregulated in multiple myeloma, which subsequently affects mitophagy, disrupts mitochondrial homeostasis, and promotes tumor progression.^[47]^ Similarly, Lesage et al demonstrated that VPS13C downregulation or mutations impair mitochondrial function, leading to the activation of PINK1/Parkin-dependent mitophagy and potentially promoting α-synuclein aggregation, ultimately accelerating the progression of Parkinson’s disease.^[48]^ MAP1LC3B (Microtubule Associated Protein 1 Light Chain 3 Beta) is a member of the LC3 (Light Chain 3) protein family, serving as a key effector in the autophagy process. It facilitates autophagosome formation, maturation, and lysosomal degradation through the conversion of LC3B-I to LC3B-II.^[49]^ MAP1LC3B plays a crucial role in neurodegenerative diseases, cancer, metabolic disorders, and immune regulation. Chiricosta et al reported that Moringin downregulates MAP1LC3B, PINK1, and other autophagy-related genes, thereby inhibiting mitophagy, reducing OS, and preventing apoptosis, which may contribute to alleviating periodontal disease-related tissue damage.^[50]^ Our study revealed that VPS13C and MAP1LC3B are significantly upregulated in periodontal disease tissues compared to healthy tissues. Nomogram, immune pathway, and immune infiltration analyses demonstrated that their expression levels are positively correlated with the risk of PD and immune activity. This phenomenon has not been previously observed in neurodegenerative diseases and multiple myeloma, suggesting 2 possible explanations. One possibility is that the upregulation of VPS13C and MAP1LC3B in periodontal disease may inhibit mitophagy, thereby exacerbating OS and leading to further tissue destruction. Alternatively, VPS13C and MAP1LC3B may enhance excessive mitophagy, resulting in immune cell activation, chronic inflammation, and ultimately exacerbated periodontal tissue damage. These 2 mechanisms are not necessarily mutually exclusive and may function at different stages of periodontal disease or in different cell types. Further research is needed to explore the context-dependent regulatory mechanisms of VPS13C and MAP1LC3B across different diseases and to elucidate their precise roles in mitophagy and inflammatory responses. This study reveals the high-expression of VPS13C and MAP1LC3B in periodontal disease and their potential association with mitophagy, suggesting that they may serve as novel targets for regulating chronic inflammation and mitochondrial homeostasis.

Despite the valuable insights provided by this study, several limitations should be acknowledged. First, this study primarily focused on the role of mitophagy in soft tissues, without extensively investigating its potential impact on bone formation and resorption. Future research should incorporate osteoblasts, osteoclasts, and related signaling pathways to further elucidate its underlying mechanisms in bone remodeling. Moreover, due to experimental constraints, this study validated the differential expression of core genes in periodontal and healthy tissues only at the mRNA level using qPCR, lacking protein-level verification. Therefore, further studies should incorporate Western blot and immunohistochemical analyses to confirm protein expression patterns, while employing cellular and animal models to systematically investigate the functional roles of these core genes in the inflammatory microenvironment through gene knockout, overexpression, and functional assays.

## 5. Conclusion

This study identified BNIP3L, VPS13C, CTTN, and MAP1LC3B as core mitophagy-related genes associated with PD, and highlighted the pivotal roles of monocytes/macrophages and endothelial cells in disease progression. These findings provide new insights into the pathogenesis of PD and offer a theoretical foundation for mitophagy-targeted diagnosis, biomarker identification, and precision therapy.

## Acknowledgments

We thank all the participants in this study. We acknowledge the use of ChatGPT (OpenAI) for language editing assistance during the preparation of the manuscript. The authors are fully responsible for the content and interpretation of the results.

## Author contributions

**Conceptualization:** Qisheng Hu, Yongheng Zhang, Huawei Ming, Zongyi Yuan, Fangyuan Chen, Wenjie Hao.

**Data curation:** Qisheng Hu.

**Formal analysis:** Qisheng Hu.

**Investigation:** Qisheng Hu.

**Methodology:** Qisheng Hu, Huawei Ming, Zongyi Yuan.

**Resources:** Xiaoyao Tan, Xingan Zhang.

**Software:** Qisheng Hu, Fangyuan Chen, Wenjie Hao.

**Supervision:** Xiaoyao Tan, Xingan Zhang.

**Validation:** Qisheng Hu, Yongheng Zhang.

**Writing – original draft:** Qisheng Hu.

**Writing – review & editing:** Qisheng Hu.

## Supplementary Material


